# Infant Care Practices among Resettled Refugee Mothers from East and Central Africa

**DOI:** 10.3390/children7060063

**Published:** 2020-06-17

**Authors:** Lauren R. Bader, Jennifer Ward, Hillary N. Fouts, Julia Jaekel

**Affiliations:** 1 Institute for Advanced Study in Toulouse, University of Toulouse Capitole, 31080 Toulouse CEDEX 06, France; lauren.bader@iast.fr; 2 Institute of Agriculture, University of Tennessee, Knoxville, TN 37996, USA; jward38@utk.edu; 3 Department of Child & Family Studies, University of Tennessee Knoxville, Knoxville, TN 37996, USA; hfouts@utk.edu; 4 Department of Psychology, University of Tennessee Knoxville, Knoxville, TN 37996, USA; 5 Department of Psychology, University of Warwick, Coventry CV4 7AL, UK

**Keywords:** refugee, infancy, parenting, Sub-Saharan Africa

## Abstract

Refugees often parent under extreme circumstances. Parenting practices have implications for child outcomes, and parenting in the context of refugee resettlement is likely to be dynamic as parents negotiate a new culture. This study examined African origin mothers’ infant care values and practices related to feeding, carrying, and daily activities following resettlement in the Southeastern region of the U.S. Ten African origin mothers were asked about their infant care practices through semi-structured interviews. Results indicated that mothers valued breastfeeding but often chose to use formula as a supplement or instead of breastfeeding. In addition, participants valued carrying their infants close to the body but used equipment such as strollers. Mothers expressed that perceptions of American culture and rules, social support, interactions with community agencies, and the need to engage in formal employment were factors that influenced their infant care practices.

## 1. Introduction

A 2019 report indicated that over 70 million people have been displaced worldwide and approximately 25.9 million people were living as refugees who were forced to flee their homes due to persecution based on race, religion, nationality, or membership in a particular social or political group at the end of 2018 [1]. Refugees often live in camps and other inhospitable living conditions for years before being resettled [2]. Daily life after a refugee’s initial flight from their home country is often characterized by limited access to resources, hunger, few job opportunities, and continued violence [1]. Resettlement also comes with potential difficulties for families, including cultural and language differences within the host country and psychological stress in both parents and children stemming from resettlement stress [3,4].

The U.S. refugee resettlement program is over three decades old, and the last decade has seen approximately 70,000–80,000 refugees resettled annually from many different countries as part of a global humanitarian effort to alleviate suffering and offer new opportunities to those who have been the victims of persecution [5]. In particular, arrivals from Africa to the U.S. have increased from 9670 in 2009 to 20,400 in 2019. Of those, a marked increase in arrivals from the Democratic Republic of the Congo and other East and Central African countries has occurred due to ethnic conflict and war within those countries [6]. The decades of resettlement from East and Central Africa mean that social and health systems are serving thousands of resettled refugees.

Given the magnitude of displacement and possible subsequent adverse life circumstances, it is worth examining the impact that forced migration might have on parenting and child well-being. In addition to potential traumatic experiences that may have preceded their migration, the resettlement process is often characterized by stress in learning and understanding a new culture, which is likely to have an impact on families and their functioning [7]. With regard to this increased stress, migration may have a deleterious impact on health, especially when cultural orientations are mismatched, and contextual demands differ from life prior to moving [8,9]. This paper explores how recently resettled mothers care for their infants by asking about caregiving practices and beliefs. For example, we asked mothers how they fed their infants and whether or not these feeding practices would have been different if their child had been born in their country of origin. Refugees from many different parts of the world resettle in the U.S., but this paper focuses specifically on those from East and Central Africa including Burundi, Rwanda, and the Democratic Republic of the Congo who have settled in the Southeastern U.S. The context within which refugees live is likely to inform their parenting and the way in which they modify their practices in movement between cultures. For instance, Stewart and colleagues [2] outlined challenges and barriers perceived by African refugees who live in Canada, which often include stress, marital discord, past trauma, poorly coordinated services, and discrimination. Migrant mothers also often have expectations akin to their country of origin, including in pregnancy and childbirth [10]. Moreover, the refugee experience is often characterized by stress, persecution, conflict within the community, and a lack of support [11], with a significant impact on health outcomes such as depression, pain, and poor health in general [12]. These complicating markers of distress are likely to impact parenting and in turn, infant and child development.

### 1.1. Developmental Niche Framework

The developmental niche provides a framework for studying and understanding child development within context and includes customs of childcare, the physical and social settings of daily life, and parenting beliefs, which interact to situate the early environments in which children develop [13]. Cultural variations in customs of childcare include how and for how long infants are held and carried [14,15], when and for how long infants are breastfed [16], and who takes care of young children [17]. The physical and social settings of the environment can affect where children sleep [18], who children play with [19], and what activities children engage in (e.g., household work) [20]. Parenting beliefs can include how mothers interpret and respond to their infants’ emotions [21]. The developmental niche of young refugee children may be altered as a result of the migration process. The following customs of childcare, parenting beliefs, and physical and social settings are of particular interest when examining how young children’s developmental niches may be affected by migration due to their prevalence in East and Central Africa—the origin of our participants.

### 1.2. Customs of Childcare

#### 1.2.1. Breastfeeding

Infant feeding choices can have lasting health consequences. Although breastfeeding is the biological norm for human offspring, the majority of infants in the U.S. receive formula. Studies have shown that formula-fed infants have a higher risk of infections and of becoming obese than breastfed babies [22,23,24]. Formula compared to breastfeeding is known to increase the risk of infants developing acute infections [25], dying from Sudden Infant Death Syndrome (IDS) or developing diabetes, obesity, and asthma [24,26,27]. As a result of these well-established disadvantages of formula feeding for mothers, babies, and the community as a whole, breastfeeding is recommended by the American Academy of Pediatrics [24], the Academy of Nutrition and Dietetics [27], and the World Health Organization [26] as the exclusive mode of feeding from birth to six months. In Sub-Saharan Africa (SSA), breastfeeding has been associated with a reduction in infant mortality [28,29].

Even though parenting throughout SSA is marked by cultural diversity, there are patterns of infant care among these populations that are distinct from dominant patterns of infant care in the U.S. In particular, data collected on infant feeding among groups in Eastern and Southern Africa have shown relatively high rates of breastfeeding initiation and duration [30]. Although extensive breastfeeding is a common practice throughout many parts of Africa [31], patterns of breastfeeding and cultural beliefs about breastfeeding often differ by ethnicity [16,32,33]. Weaning practices vary in Sub-Saharan Africa. In a study on rural Kenyan toddlers, 64% of 12–36-month-olds were fully weaned [34]; the initiation of weaning began within 12 to 18 months among 43.9% of Nigerian infants [35]; 21.9% of rural Tanzanian infants were found to be weaned prior to two years [36]. Gibson-Davis and Brooks-Gunn [37] found that with each additional year living in the U.S., immigrant mothers had a 3% decrease in the odds of breastfeeding at 6 months. In contrast, mothers in high-income countries tend to feed their infants for a shorter duration than mothers from low and middle-income countries [29], and only 24.9% of infants in the U.S. are exclusively breastfeeding at six months of life [38]. Thus, it’s possible that mothers’ weaning was associated with time in the U.S.; however, because mothers in this study gave birth to the focal child in the U.S., we cannot determine how immigration impacted weaning.

#### 1.2.2. Infant Carrying and Skin-to-Skin Contact

Carrying infants for long periods of time is common throughout many parts of Africa [39,40], is linked to shorter durations between breastfeeding bouts [15], and is positively associated with breastfeeding frequency in Italy [41]. Infant carrying is also associated with cultural beliefs in many SSA foraging societies [40]. Compared to infants in the U.S., Ngandu and Aka infants (central Africa) have been found to be held for a significantly greater percentage of time [42]. Similar to breastfeeding, infant carrying, and skin-to-skin contact has benefits for infants’ health and development. Carrying infants close to the body can stabilize infant temperature and improve bonding and positive interactions [43,44,45]. Skin-to-skin contact is especially beneficial to preterm infants and has been linked to reduced stress and anxiety among parents of preterm infants [46,47].

#### 1.2.3. Daily Practices

Daily practices including singing to infants are practiced in many cultures and may help support secure attachment relationships between mothers and their infants [48,49,50]. Infants also appear to be more attuned to their mothers when they sing to them compared to when they speak to them [49]. Similarly, baby massage may help to calm fussy or colicky infants [51] and promote growth in preterm infants [52]. Keller [53] discussed this type of parenting practice that includes touch and massage within the *stimulation context*. For instance, in some cultures such as the Nso in Cameroon, parents’ use of touch to stimulate infants has been shown to promote motor development [53].

Mothers in many parts of SSA have been found to be highly responsive to infant fussing and crying and other signs of distress, often practice prolonged breastfeeding (i.e., breastfeeding beyond one year), and frequently carry babies on their body using wraps and slings [14,16,54,55].

#### 1.2.4. Impact of Migration on Customs of Childcare

Parenting practices include a wide array of behaviors beyond the scope of a single study. This study focuses on infant feeding, carrying, and daily routines as described by refugee mothers from SSA.

Little is known about how infant care practices change with forced migration or how these practices change over time in a new country. Commonly known as the immigrant paradox, refugees and immigrants have many strengths that contribute to well-being and may be protective of health disparities common among racial minorities and others with low social status [56]. For example, among a sample of immigrant women living in the U.S. and representing most world regions, 83% chose to breastfeed, which resulted in significantly better health for infants in terms of weight, hospital admissions, and general health [57]. However, the proclivity to breastfeed may change over time via the process of acculturation where breastfeeding rates decrease as the length of time in the U.S. increases [37]. Furthermore, immigrant mothers living in the U.S. are typically more likely to breastfeed than to use milk substitutes and to wean their children later compared to US-born women [37,58]. Women from Africa who were living in Canada as asylum-seekers, refugees, or non-refugee immigrants were more likely to be breastfeeding at 16 weeks postpartum compared to those born in Canada and other groups [59]. Furthermore, the length of time residing in the United Kingdom among immigrant mothers was related to a decrease in breastfeeding when compared to first- and second-generation mothers [60]. Accordingly, research shows that refugee mothers may use infant care practices that benefit infant health amid economic disadvantage; however, refugee mothers’ infant care practices, specifically breastfeeding, have been shown to decrease over time following immigration to Western countries [60]. In addition to infant feeding, carrying and other parenting practices may change after resettlement in a new country, but existing research is scant, and it is not well known how certain practices change with migration.

#### 1.2.5. Parenting Values and Beliefs

Parenting values are shaped by shared cultural beliefs or foundational schemata within societies [61]. Foundational schemata such as egalitarianism, autonomy, and independence [61] can shape parenting values and practices and have been found to be associated with breastfeeding [16,32], weaning [62,63], and responses to infants’ emotions [21]. For example, Fouts and colleagues [16] argued that Bofi and Aka forager (Central African Republic) children’s control over breastfeeding, including how often they fed, was related to the Aka’s core cultural vale of personal autonomy.

#### 1.2.6. Impact of Migration on Parenting Values and Beliefs

Although norms in a home country are well-understood and cultural scripts are easier to follow, refugees must find new ways to adapt their lives after moving to a new culture. Thus, African origin mothers negotiate their normative parenting behaviors and the demands of a new living environment [64]. The process of this adaptation has traditionally been studied through the lens of acculturation measured by the degree to which newcomers adhere to a new country’s norms and the length of time spent in that new country [60]. A less linear approach to how immigrants negotiate living in a new culture is the idea of cultural translation whereby people hold on to values of their country of origin while simultaneously adopting new strategies in complex ways [65]. Both views provide a useful lens with which to view the phenomena of infant care after migration.

Hohl and colleagues [9] conducted interviews with Hispanic women mostly born outside of the U.S. but currently living in the state of Washington and found that they valued breastfeeding and its health benefits, but they were embarrassed to breastfeed in the U.S. The participants perceived that breastfeeding was valued less among the American public and subsequently, many decided on a combination of formula and breastfeeding or solely formula fed their infants [9]. Similarly, Schmied and colleagues [8] found in a meta-ethnographic review that immigrant mothers from many different countries who had settled in Australia, the United States, England, and Canada reported feeling tension between their beliefs and their perceptions of their new culture and its values in regard to breastfeeding, which resulted in embarrassment and the early cessation of breastfeeding.

#### 1.2.7. Physical and Social Settings of Childcare

In the U.S. in 2018, 60% of children lived in a household with two-biological married parents [66]. Around 30% of children have lived with a grandparent at some point, often in a three-generation household [66]. Among immigrant adults in the U.S. in 2017, around 61% included married parents compared to 48% of adults born in the U.S. [66]. In 2018, poverty rates varied by ethnicity with non-Hispanic White children at 12% compared to 30% for Black children [66]. Thus, the make-up of U.S. families varies by ethnicity and immigrant status and is dynamic depending on economic circumstances such as the Great Recession [66].

Children are raised in many different physical and social contexts that include variations in who cares for children and where. For example, in many countries in SSA, infants’ siblings are responsible for much of their daily care [67]. Among Aka foragers in Congo, besides mothers, extended family may also be responsible for feeding children [68]. The immigration of refugee mothers from SSA to the U.S. is sure to transform usual physical and social settings of childcare, especially among mothers who will no longer have access to the family support system they could rely on in their countries of origin [69]. In addition, the need to work for pay in the U.S. could alter childcare, as mothers may need to work outside of the home and enroll their children in formal childcare. Work outside of the home could also change mothers’ feeding behaviors if she will be away from her child for periods throughout the day.

#### 1.2.8. Impact of Migration on the Physical and Social Settings of Childcare

The physical and social settings of children in the U.S. are characterized by stark differences depending on families’ economic and sociocultural backgrounds. Accordingly, Vesely [69] found that many immigrant mothers from Africa were particularly interested in sending their young children to early childcare centers in order to provide opportunities to spend time interacting with other children, which was similar to how children would have spent their days in their countries of origin [69]. Mothers also cited the need for early childcare centers because of the stress they felt without family support with childcare that they would have received in their country of origin [69]. Missal and colleagues [70] found that Somali immigrant mothers discussed the loss of their traditional support system in the postpartum period in interviews about their childbirth experiences in the U.S. Mothers discussed the desire to build relationships with nursing staff in order to receive advice on breastfeeding and childcare [70]. Refugee mothers have said that health care providers were sources of both informational and emotional support in the perinatal period [71].

Refugee mothers face multiple stressors beyond immigrating to a new country for protection, including interactions with persons who have different childcare practices and cultural beliefs regarding childcare, and new social and institutional resources surrounding childcare in their new countries. It is important to understand these experiences of refugee mothers in order to better help them navigate childcare in their new setting. The present study seeks to expand on previous qualitative research and addresses the following question: How do refugee mothers from East and Central Africa in the U.S. describe their experiences and practices of infantcare in the U.S.? We expected that mothers would express challenges related to their transition to living in a new country and describe their infant care practices as somewhat modified from how they describe their behavior in their home country. A lack of research on the particular infant care practices addressed in this study limited the ability to formulate more specific hypotheses about parenting behavior.

## 2. Methods

### 2.1. Data Collection

Criterion sampling [72] was used to recruit mothers who were first-generation African refugee mothers who had recently emigrated to the U.S. from SSA through the United Nations High Commissioner for Refugees (UNHCR) resettlement process. All but one participant were refugees resettled in the U.S. as part of the UNHCR refugee resettlement program. This mother self-identified as an immigrant rather than a refugee. However, she indicated that it would be difficult for her to return to her home country of the Democratic Republic of Congo (DRC) due to violence and the fact that her home was burned down. Her responses to our interview questions demonstrated that she shared life experiences typical for refugee mothers from the DRC and Burundi. Thus, we analyzed this mother’s interview responses along with the responses from the refugee mothers. The majority of recent refugees from SSA living in the catchment area of this study are from Burundi and the DRC. Thus, we expected most of our participants to identify as Burundian or Congolese. According to the Tennessee Office for Refugees [73], the DRC had the most refugees resettled in Tennessee in 2019. Mothers were recruited through a local non-profit organization that provided English language lessons for refugees in a mid-sized city in Tennessee. Mothers were eligible to participate if they had at least one child who was 24 months of age or younger and if they were a refugee from a SSA country. Mothers were recruited through word-of-mouth and snowballing, where participants would suggest other people in their English classes to contact for an interview [74]. Recruitment flyers were displayed throughout the community center building, where the English language lessons took place, public locations, and distributed by staff at the local refugee resettlement service. Members of the research team visited English classes in order to invite participants to complete an interview. Interested participants had the choice to complete the interview immediately on site or schedule an interview in their own home at a later time. Institutional review board (University of Tennessee Knoxville IRB-16-03052XP) approval was obtained prior to data collection. Every participant signed an informed consent written in English and their preferred language (French, Kirundi, Kiswahili, or Swahili) after verbal explanation of the study purpose and process in their preferred language.

### 2.2. Participants

The research team conducted interviews with 10 mothers from the DRC and Burundi. Interviews were conducted between February 2016 and March 2017. Mothers were on average 29 years old (23–37, *SD* = 4.4) and had been in the U.S. for an average of 4.2 years (0.1–10, *SD* = 4.4). Focal children were on average 12.4 months of age (2–26, *SD* = 8.4) at the time of the interview (Table 1).

### 2.3. Interview Guide 

Interview questions (see example questions in Appendix A below) focused on how mothers fed, carried, and generally provided care for their children. Questions also addressed how mothers thought their practices may have changed with migration or would have been different in their pre-resettlement contexts. A trained research team member conducted interviews in the participant’s preferred language (French, Kirundi, or Kiswahili, or Swahili) with the assistance of an interpreter and documented through extensive notetaking and audio recordings [72,74]. Mothers were asked to talk about their experience caring for one child in particular who was 2 years of age or younger, which is hereafter referred to as the focal child. Each participant was given a $10 gift card for completing the interview. Interviews typically lasted 45–60 min. All participants were given the opportunity to ask questions, and if they expressed a need related to social services or immigration, the research team had a resource list available for them to use and referred them to the local resettlement agency for more help.

We utilized a qualitative research design with semi-structured interviews. The questions included four sections: Demographic Background, Feeding, Carrying, and Other Mother–Infant Interactions. The Demographic Background section included questions about migration, length of time in the U.S., family composition and living arrangements, and other general questions. The Feeding section contained questions about breastfeeding initiation, duration, supplementation, weaning, and the factors that influenced feeding decisions. The Carrying section was focused on how the mother carried the focal child and whether and how that had changed in various migration contexts and daily life situations (e.g., at church, at home, at the grocery store). The Other Mother–Infant Interactions section of the interview focused on daily care activities including play with toys, singing, and washing routines. Interview questions were checked and modified for wording, content, and cultural relevance following the first initial interviews [72]. We identified that thematic saturation [75] was reached after interviews with 10 mothers and thus, stopped following this final interview. 

### 2.4. Data Analysis

Audio files of the 10 interviews were de-identified and transcribed in English by trained graduate student research team members. After transcribing, each member of the research team did an initial review of all interviews following an iterative process [72,75]. Then, Feeding, Carrying, and Other Mother–Infant Interactions sections of the interview were coded by two team members each for the initial round of open coding using NVivo (Version 11; QSR International Pty Ltd., Melbourne, Australia, 2015). Then, the research team met and identified five main themes, and a codebook was developed based on those themes. A second round of coding was based on the developed codebook. Then, another meeting was held to determine the reliability of coding between researchers (i.e., resolving differences in coding), to decide if any new codes were needed for emergent themes, to discuss sub-themes, and to discuss relationships between themes [76]. After identifying initial themes from the coding process, the ways in which the themes connected were considered. Axial coding revealed a transactional relationship between themes [75]. The research team met and discussed the data to decide how the themes related to each other. Through this discussion, the themes of social and institutional resources were found to interact with the daily life demands in such a way as to support or inhibit the mother’s desired infant care practices. Descriptive statistics were run in SPSS (Version 25; IBM Corporation, Armonk, NY, USA, 2017).

## 3. Results

Five major themes were identified: Cultural Norms, Daily Life Demands, Social Resources, Institutional Resources, and Cultural Disconnects. Sub-themes were also identified and are described under each theme below.

### 3.1. Cultural Norms

Mothers described the ways they typically cared for their infants in terms of breastfeeding, carrying, and daily care practices. In terms of breastfeeding, all women expressed that they breastfed initially at birth, and most continued to breastfeed. Breastfeeding was the preferred mode of infant feeding both with infants born in the U.S. and those infants who were born in refugee camps. One mother indicated, “I always wanted to breastfeed”, and another mother said, “…the breastmilk is better than the formula.” One mother said, “…in my culture, everyone chooses breastfeeding.” Weaning age varied among participants including partial weaning around 3 to 4 months with the introduction of complementary food and total weaning between the ages of 8 and 18 months.

Some participants revealed that they also used formula to feed their infants in a supplementary way. More than half of participants indicated experience with using the federally funded Special Supplemental Nutrition Program for Women Infants and Children (WIC) and thus used the formula brand for which they received vouchers. Mothers were asked if they would have used formula in their home country and the majority said that they would not, either citing cost or the cultural norm to breastfeed without any use of milk substitutes. Many mothers explained that they had breastfed children born prior to the focal child.

In terms of carrying, most mothers indicated that they carried their babies primarily on their back both in the U.S. and in their home country or country of refuge in Africa (this is the country or countries that refugee mothers stayed in after leaving their country of origin prior to coming to the U.S.). Most explained that it is the norm in the countries of their birth or refuge, and that they learned how to do it from growing up around the practice and carried younger siblings in this way. For instance, one mother said, “Nobody teach us how to carry babies on the back because it is traditional. When you grow up you see. You don’t have anyone to teach you.” Another mother said, “…if you don’t want to carry your baby like that, I don’t think they will bond with you.” Wrapping the baby on the body was indicated as a primary method of holding, although most mothers talked about holding the infant in their arms and waiting until the infant had some head control around four months of age to carry on the back. In fact, one mother explicitly talked about culture when distinguishing between her practices and those of other populations, indicating that in Namibia “white women use this” (and showed the interviewer a name brand structured baby carrier), and then explained that she used a traditional African cloth to carry her baby.

### 3.2. Daily Practices

Mothers also described daily care practices that included bathing, body manipulation, and singing to their children. One mother said she is “always singing, always” to her baby and that in turn her baby “is always singing ABC even when he didn’t know how to talk.” Another mother responded to the question about singing/music saying that she sings “all the time whenever I feed her, I start to sing to her”. Bathing rituals were discussed and were similar in that most mothers bathed their infants every one to two days with some indicating that the weather had an influence on the frequency. For example, if it was too cold, one mother said the bath would only be every other day. When asked what babies need to grow well, mothers responded with a variety of answers including love, attention, and good food. One mother explained a stretching routine for the infant’s limbs and said, “if you don’t do that then her elbows will be like this [indicated inward joints], If you do that [stretching], they will be straight.” Cultural norms were inextricably linked to mothers’ infant care practices, but the daily life demands of living in a new environment with employment requirements and travel between home and grocery stores also brought up different challenges related to infant care practices.

### 3.3. Daily Life Demands

When explaining how they cared for their infants, mothers often spoke about the reasons for making their choices in terms of the demands on their new lives.

#### 3.3.1. Employment

One mother said that she nursed her child born prior to the focal child, but because he refused a bottle, she planned to train the focal child on a bottle (i.e., using formula). Given that she would have to work outside the home in the paid formal workforce, she now needed the focal child to use a bottle for when she was at work. In fact, the necessity to engage in paid formal work in the U.S. was often stated as a reason to modify breastfeeding and to use bottles and human milk substitutes. One woman said “Breastfeeding and working was kind of different, so I had to use formula. I didn’t have a choice.” Another mother said, “Yes, I stopped because I was at work and the breast was too big and it was hard that I thought if I continue to give her this one, because it takes like 7 or 9 h, I may be sick, so I stopped.” Thus, mothers reported that infant care practices, specifically breastfeeding, were influenced by their employment in the formal workforce.

#### 3.3.2. Day-to-Day Tasks

Mothers said that carrying the baby on the back was used in the U.S. for convenience. One mother explained she carries her child because it “is more comfortable than pushing him in the stroller…I can’t carry the stroller and push it… So I just take a wrap when I go to the store.” In contrast, other mothers noted that because of traveling in the car they would need to use a car seat and then “just carry him in my hands or on my back” after departing the car. The need to travel around town in cars and to walk to the grocery store were indicated as reasons for using strollers and car seats rather than using a traditional cloth to wrap the baby on the mother’s back. Being at home and the convenience of carrying the baby on their backs was recognized by some mothers as a way to help soothe the baby. One mother claimed she carried her baby on her body “so he can sleep a lot.” Overall, it appears that the demands of daily life both modified and supported core cultural practices of breastfeeding and carrying. Mothers’ social resources were reported to provide assistance with tasks related to daily life, including helping to care for children.

### 3.4. Social Resources

Participants were asked specific questions about their social networks and whether and what kinds of help they received with the demands of daily life and caring for children.

#### 3.4.1. Family

Mothers were asked about the kind of social support they had in caring for their children in the U.S. Most had relatives that were overseas in either the mother’s country of origin or the country of refuge, and a few mothers had close family in the resettlement city. However, help from the close family members wasn’t always available because of demands, such as work. For example, one participant said of her mother-in-law, “She’s working full time … so she doesn’t have time” to help with the children. Another participant had her parents and siblings with her in the U.S., but she was the only adult with her own family and therefore could not rely on either her minor siblings or her parents for help with her children.

#### 3.4.2. Friends and Neighbors

Many mothers said that they had friends and neighbors that they could rely on for help with short-term childcare but did not indicate that any of these people were involved in wearing or feeding their infants. Similar to the situation with family members, participants indicated that friends were usually working so they did not see them often. One mother described that to run errands, she asked a neighbor for help with her children. “If I want to go somewhere, I just take my kids to her then I say, ‘Can you watch my kids?’ and I’ll be back. And she will stay with them.” Another participant cited her friends as a source of moral support and instrumental support with caregiving. Specifically, the mother said other Congolese mothers help her with “Moral support. And if you need something, they can see what they can do.” Thus, her friends were also providing some instrumental support.

### 3.5. Institutional Resources

Mothers were asked about community agencies and other groups that provided help to their family, particularly in respect to caring for their children.

#### 3.5.1. Hospital

Hospital experiences, with respect to the birth of the focal child, were discussed by participants. Most indicated positive delivery experiences both in countries of refuge and locally in the resettlement city. One mother recounted a lack of support in the hospital with regard to her birthing wishes. For example, when she felt as though she needed to push, the nurse “kept saying ‘No, you need to wait, you need to wait.’” Another participant who had a positive experience chose that particular hospital as the place of delivery because she liked the doctor, explaining that “…The doctor was nice, and he kept asking me how I’m doing all the time. I like to go there.” The hospital was also a place mothers named as the original source of formula and referral to WIC.

#### 3.5.2. WIC Program

When mothers discussed the use of formula, they described WIC as the provider of their formula vouchers. One mother said, “…They [the hospital] tell me to go to the WIC so they can give me the vouchers, so I was on low income.” Mothers indicated that they had to choose the formula that was named on the WIC voucher and thus had little power in deciding which brand of formula to use. This mother said, “I choose that because for the first time at the hospital, that’s what they gave me.” Noticeably absent from interviews was any mention of lactation support from WIC, although that is a known component of the program for postpartum mothers (U.S. Department of Agriculture WIC Breastfeeding Support). Although not mentioned as specifically provided by WIC, one mother talked about the breast pump she was given and how that allowed her to work and pump breastmilk for her baby while she was away.

#### 3.5.3. Childcare

Mothers also cited childcare programs as a resource for help in caring for their children and meeting their need to work outside of the home. Mothers’ references about childcare were related to the themes of employment and neighbor and friends as resources. One participant said, “I used to take my kids to daycare but because now I changed shifts, I do not take them no more.” The need to work and the changes in work modified how childcare was used. When childcare was mentioned, it was most often in the context of neighbors, friends, and other family members. When talking about a neighbor who came from the same camp in Congo, a participant said, “if I were to go somewhere, I just take my kids to her…and she will stay with them.”

### 3.6. Cultural Disconnects

Participants described their perceptions of common rules and values in the U.S. as a reason for changing their infant care practices such as bottle feeding in public, carrying infants in car seats, and leaving infants with other caretakers. Most mothers acknowledged a perception that mainstream infant care practices in the U.S. were different than those they would use in their home country. So, when mothers talked about their culture and their values, they also often described the mainstream values in the U.S. as being in opposition or conflict to those of their home country. One participant described her cultural values in opposition to those of the U.S. by describing that in her country, mothers wear their babies on the back to “try to make the bond between the mom and the child, they connect. But here it is not like that.”

One mother believed that “the American law requires to feed the baby breastfeeding and formula too.” Although this was an extreme view, many mothers talked about being warned by members of their community prior to migration that they would have to feed their babies under a cover or with a bottle in public in the U.S., unlike what they were used to in their home country or country of refuge. One mother said, “Americans cannot really breastfeed in front of everyone in the room”. Another mother indicated it was the rule to feed the baby in intervals of 20 min at the hospital but that at home she would “feed her all the time” or on demand. A couple of mothers talked specifically about the expense of formula in their countries of refuge and how it was inaccessible. One mother said, “If I were rich, I would use formula,” about formula use in her previous country. The availability of WIC formula vouchers meant that they could use formula without financial barriers. Another participant described that, “If you don’t have the money, you can’t feed the baby the same way. If you don’t have just you cannot give the formula, just give whatever you have.” In other words, formula was not a viable option in the countries from which they came.

The need to use the car or bus to travel around the city meant many mothers needed to use equipment previously unfamiliar to them such as strollers. A participant explained the change in carrying by saying “Yes, in general, something changed, because when I need to go somewhere, we get in the car. I put him in the car seat.” While work typical of life in their home country or country of refuge would allow for close and extended proximity to the baby, many mothers talked about the need to work and be away from their children, thus modifying their carrying practices. For instance, two mothers described how women who “work in the fields… carry their baby on the back.” One participant explained, “If you are farming, you put baby on the back, because the baby can’t be far away from you.” Another mother said that working and breastfeeding for her now was incompatible, “So I always wanted to breastfeed, but breastfeeding and working was kind of different, so I have to use formula, I didn’t have a choice.” Despite changes in work environments, many mothers said they still carried their babies on their back in the U.S. at home, during chores, and during outings.

### 3.7. Connections Between Themes

Cultural norms described in interviews included high rates of breastfeeding and carrying on the back. Interview responses confirmed that these practices were commonplace among the participants and the participants also perceived them to be culturally commonplace for other women in their home country and country of refuge. However, in the context of the U.S., women had access to various resources that supported or modified their engagement in these practices. For instance, employment outside of the home meant that mothers used milk substitutes more often than they said they would in Africa. Help from neighbors, friends, and other family members were important sources of support to complete those daily life demands such as work and chores, but they also meant that women used more milk substitutes in bottles rather than breastfeeding. Even the anticipation of working outside the home compelled some participants to adjust their breastfeeding practices and preemptively use a bottle.

The mothers’ perceptions of available resources interacted with their cultural values and the way they perceived the values of their new home. They modified their typical breastfeeding practices, leading to partial reliance on formula because women were expected and needed to get a job outside of the home, had access to inexpensive formula, and perceived the cultural norms in the U.S. as valuing modesty and discouraging public breastfeeding. Mothers said that for various reasons, including limited access to resources in their country of refuge and varying cultural norms, that they behaved differently in the U.S. than they would have in refugee camps or in their country of origin. Social resources such as childcare from neighbors allowed for mothers to engage in formal work and household chores. Institutional resources such as WIC provided free formula vouchers to those who qualified resulting in modified breastfeeding behavior. Those mothers who did not receive such support, education, or equipment necessary to express their milk articulated that they effectively had no choice but to use formula. Lactation support is an initiative of WIC (U.S. Department of Agriculture WIC Breastfeeding Support); however, only one mother said she received the support and resources necessary to pump breastmilk at work. In other words, in our study, resources influenced the way mothers fed and cared for their infants despite their values.

## 4. Discussion

Mothers interviewed in this study indicated that they modified their infant care behaviors from what had been normative in their home country following immigration to the U.S. Results show that mothers made modifications particularly in terms of feeding and carrying, and those choices were connected to the demands of living in a new country, perceptions of cultural values and beliefs about parenting in the U.S., and their interactions with and support from social and institutional resources.

Infant care practices are rooted in cultural beliefs [13,32,62]; however, this study sheds light on why and how practices can change as a result of migration. A mother living in a new culture may modify her traditional parenting practices as a function of exposure to new cultural norms, the demands of living in a new place, and the types of resources available to her. Breastfeeding and carrying were main topics in the interviews and discussed most explicitly in terms of change in behavior. Consistent with literature examining breastfeeding and acculturation [37], mothers indicated that they breastfed less often than they would have in their home country or previous country of refuge, and reported using bottles, formula, and other equipment in the U.S. Furthermore, stressful circumstances can undermine mothers’ caregiving abilities by altering her ability to continue breastfeeding [77].

Even though mothers rarely mentioned the nutritional benefits of breastfeeding, they cited that it was good for the baby in terms of attachment and bonding. Moreover, they explained that human milk substitutes were expensive and hard to access in their country of refuge. Given their new context, perceived differences in cultural values and the low-cost availability of formula, many mothers reported to use formula more than they would have in Africa. Daily life demands, such as the need to get a job in the U.S. to pay bills, were among the most cited reasons to use a bottle and formula when the mother had to be away. In contrast, the work most mothers talked about doing in their home country (i.e., farming) was compatible both with breastfeeding and carrying babies on the body. This finding is consistent with other qualitative work that has found that perceived conflict in cultural values and apparent stigma of breastfeeding in the U.S. influence immigrant women to breastfeed less and use formula more [8,9].

Carrying practices were modified slightly to incorporate car seats and strollers and most often to accommodate transportation in cars. Some mothers said that using traditional African wraps was convenient and easier than equipment such as a stroller. However, several mentioned that the need to travel by car, rather than walking, meant that they carried their babies less than they would have in Africa. Mothers described the cultural value and longevity of carrying babies on the back with wraps by explaining that they didn’t really learn to do it, but that they just knew and had always seen babies carried this way. One mother also said that carrying her baby in this way helped the infant to bond, which could be associated with a cultural custom or foundational schema in her country of origin. For example, Hewlett and colleagues [40] noted that the prolonged holding of infants in many SSA foraging societies could be related to the development of a trusting and accepting worldview. Furthermore, carrying infants in a soft baby carrier is associated with infants’ secure attachment among Hispanic and Black mother–infant dyads in the U.S. [78]. Although carrying babies on the body is practiced in the U.S., the way in which the participants carried their babies was by using “traditional cloth” and not in structured carriers more commonly used in the U.S. Additionally, participants exclusively carried on the back whereas carrying in the front using soft-structured carriers is commonly practiced by parents in the U.S [79]. Mothers carried babies using a wrap both in the home and out in the community, but work in the U.S. was not compatible with this type of baby carrying as it is with small-scale subsistence farming in Africa. Although the effects of changes in carrying practices from carrying on the body to the use of more equipment such as strollers has not been studied, carrying babies on the mother’s body may have positive physical and emotional health benefits that should be supported. Further studies could elucidate how changes in carrying practices may impact responsivity to infant needs and prolonged close body contact, which are known to have benefits for infant development [43,80]. Since participants perceived that few mothers in the U.S. carried their babies by wrapping/holding, it is possible that they were influenced by this observation and opted for carrying children in strollers or car seats rather than on their bodies.

Social and institutional resources appeared impactful on the physical and social settings of childcare and how mothers chose to care for their infants. Mothers often cited the expense and lack of availability of formula in their country of refuge as a reason that they typically breastfed there. However, that practice was subject to change when mothers encountered easy access to formula in the U.S. through WIC and a need to be away from their infants because of formal work. Moreover, some women perceived formula use to be preferred or even required by people in the U.S. Migrant mothers have appeared to be unable to argue for their traditional cultural practices when provided with contrasting instruction from health care providers [10]. This may help explain some of the reasons why previous quantitative research shows a decline in breastfeeding rates with increasing number of years living in Western countries in samples of immigrants [37,59]. Studies have suggested that WIC supports breastfeeding and delivers information regarding the benefits of breastfeeding to mothers [81,82]; however, other studies have identified a negative relationship between mothers receiving WIC benefits and breastfeeding initiation and duration [83].

Many mothers also mentioned that they did not have the same social support system with childcare that they would have had in their country of origin. Mothers mentioned that most of their family was still in their country of origin or living in another part of the U.S. One mother said that even though she had friends to help out with childcare, they worked most of the day. This is consistent with qualitative meta-syntheses that found that migrant and refugee women missed the abundant care provided by extended family following the birth of a child [10,71]. Thus, it appears that mothers’ childcare options were limited compared to what they would have had in the DRC or Burundi with extended family and friends living close by to help out with raising children.

### 4.1. Implications for Policy and Programs

Resettlement and humanitarian agencies, social service providers, policymakers, educators, and health providers in the U.S. may serve refugee families best by understanding the particular ways in which the stress of displacement affects family dynamics and relationships [2]. For example, given that refugees and other immigrants are culturally inclined to breastfeed [29], service providers may not target them for breastfeeding promotion efforts. New Somali immigrant mothers in the U.S. had this exact concern [70]. Missal and colleagues [70] explained that the nursing staff may have assumed that the new Somali mothers did not require any help because the practice of breastfeeding is so commonplace in their home country.

Those interested in promoting breastfeeding and protecting the health of vulnerable populations should understand the factors that influence the decline in breastfeeding behavior such as the need to engage in paid work outside the home and the perceived cultural stigma around breastfeeding in the U.S. It may be beneficial for health providers to make concerted efforts to talk to mothers who are originally from SSA about breastfeeding in light of this data. Counselors and other providers at WIC can incorporate an understanding of cultural values into their training to determine what food packages are most ideal for these mothers and to emphasize breastfeeding as a strength. Despite the well-documented health benefits of breastfeeding, participants in this study did not cite health benefits as reasons to breastfeed. This indicates a potential area for explicit teaching to mothers, even among those who breastfeed. This could potentially add value to the practice and help mothers to overcome potential cultural and practical barriers to breastfeeding in the U.S.

Resettlement and other community agencies should collaborate to ensure that refugee mothers have access to resources that support the choices they want to make to care for their children, including navigating the insurance system that would allow them a free breast pump, training and orientation to breastfeeding in the workplace, and safety training in milk storage and handling. Resettlement agencies and others who interact with recently resettled refugees should seek to understand and subsequently support mothers in continuing their valued infant care practices including infant carrying. By recognizing their infant care practices as strengths, mothers may feel more supported and be more inclined to keep the practice, and this support may counteract stigma that mothers encounter.

### 4.2. Limitations

As with all research where the researchers and participants speak different languages, the quality of the data depended heavily on the accurate language of interpreters. Even though the research team believes the findings are credible and reflective of the participants’ thoughts and feelings due to similar findings on the concerns of SSA mothers who have immigrated to the U.S. [70], certain words and meanings are subject to being lost or misinterpreted. However, three different interpreters were used, so the consistency of emergent themes reinforces the fidelity of the findings to the meaning the participants intended to convey. Most participants were also relatively new arrivals and thus had not experienced motherhood in the U.S. for a long period of time. Incorporating individuals who had been in the U.S. longer might show how infant care practices change with time. Furthermore, our interview questions did not allow us to probe further regarding how mothers came to their decisions when navigating various infant care practices. This would be a good area to follow up with when asking mothers’ to describe who they rely on for information and opinions when making decisions.

## 5. Conclusions

Infant care practices are culturally ingrained and contextually dependent. Health behaviors that are a cultural strength of mothers originally from SSA may be subject to change with time in the U.S., including breastfeeding and carrying infants on their body. This is the case especially considering that the context of resettlement for refugees is one of dynamic change and stress that can impact children and families [2,11]. Social service providers and medical services can combat perceived cultural conflict and the demands of daily life by supporting mothers and connecting them to helpful resources. The opportunity to support cultural strengths such as the tendency to breastfeed is one that should be considered a public health priority. In order to understand the mechanisms behind reduced breastfeeding rates or changed infant care practices after migration to the U.S. and other Western countries, we must understand how women care for their infants after emigrating and why they make those choices.

## Figures and Tables

**Table 1:** Demographic characteristics of participants (*N* = 10).

Characteristic	Categories	*N* (%)	Mean (*SD*)	Range
Age of mother (years)			31.13 (2.45)	23–44
Age of infant (months)			13.60 (2.76)	2–27
Focal child gender	Male	6 (60.00)		
	Female	3 (30.00)		
	Undisclosed	1 (10.00)		
Country of birth of focal child	Home country	1 (10.00)		
	Country of refuge	3 (30.00)		
	United States	6 (60.00)		
Current mode of feeding	Exclusive breast	2 (20.00)		
	Breast and table food	3 (30.00)		
	Formula and table food	1 (10.00)		
	Weaned	4 (40.00)		
Mode of feeding until 6 mos.	Exclusive breast	5 (50.00)		
	Breast and formula	5 (50.00)		
Mode of carrying	Arms	1 (11.11)		
	Back	7 (77.78)		
	Equipment	1 (11.11)		
Country of origin	DRC	5 (40.00)		
	Burundi	5 (60.00)		
Employment	Employed	2 (20.00)		
	Unemployed	8 (80.00)		
Spouse Employment	Employed	6 (66.67)		
	Unemployed	3 (33.33)		
Length of time in US (years)			3.36 (1.21)	0–9
Length of time in study region (years)			1.56 (0.54)	0–5
Number of camps lived in			1.20 (0.34)	0–4
Length of time in camp (years)			14.75 (3.37)	3–30
Number of children delivered			3.70 (0.82)	1–8
Number of children living			3.00 (0.65)	1–7
Number of children in household			3.00 (0.73)	1–7

*Note*. *SD* indicates standard deviation. DRC: Democratic Republic of Congo.

## Appendix A. Sample Questions Included in Semi-Structured Interview Guide

1. Please tell me a little bit about your migration history.
2. Where in Africa have you lived after leaving your home country? For how long?
3. Did you stay in a camp in Africa? If yes, what was the name of the camp you stayed in in Africa? How long were you in the camp?
4. When did you arrive in the U.S.?
5. What language do you and your spouse speak with your family?
6. Are you and your spouse employed in a job?
7. Please tell us all the jobs you and your spouse do.
8. How many hours do you and your spouse work in one week?
9. How many children have you given birth to?
10. How many of these children were born in Africa?
11. How many of these children were born in the U.S.?
12. What city did you give birth to the children born in the U.S.?
13. Why did you decide to give birth in that hospital/birth center/other location?
  - How was your experience giving birth there?
  - Were you able to communicate your wishes with the nurses/doctors/midwives?
  - Can you give me an example?
14. Do you have friends or family members that help you with childcare?
15. Do you have friends that help you with childcare?
16. In the way you talked about your friends helping, are there any community organizations that help you in any way?
17. How did you feed your baby at birth?
18. Have you been breastfeeding? For how long? How has your feeding changed over time?
19. How do you currently feed your baby? (breast, bottle or solid food, or combination?)
20. Did you have any difficulties with breastfeeding?
21. Did anyone give you formula for free?
22. Do you use a particular brand of formula? Why or why not?
23. Do you use the same brand of formula every time?
24. Would you feed your baby differently in your home country?
25. Is carrying babies on the body a typical parenting practice in your home country?
26. Why do mothers carry babies on their body?
27. Do you think that all mothers should carry their babies on their body?
28. What material do you use to carry your baby (wrap/sling/back or front/etc.)?
29. Do other family members or friends wrap your baby on their body when taking care of the baby?
30. In what situations do you usually carry your baby? (ex: at home, while traveling, etc.)
31. How often do you carry your baby on your body? Every day?
32. Has the way you carry your baby changed since you have been in the U.S.?
33. Tell me about a good experience with carrying the baby on your body? (ex: someone complimented their wrap or sling in the grocery store).
